# Doxorubicin Paradoxically Ameliorates Tumor-Induced Inflammation in Young Mice

**DOI:** 10.3390/ijms22169023

**Published:** 2021-08-21

**Authors:** Ibrahim Y. Abdelgawad, Marianne K. O. Grant, Flavia E. Popescu, David A. Largaespada, Beshay N. Zordoky

**Affiliations:** 1 Department of Experimental and Clinical Pharmacology, College of Pharmacy, University of Minnesota, Minneapolis, MN 55455, USA; abdel217@umn.edu (I.Y.A.); grant032@umn.edu (M.K.O.G.); 2 Department of Pediatrics, Genetics, Cell Biology and Development, Masonic Cancer Center, Medical School, University of Minnesota, Minneapolis, MN 55455, USA; pope0014@umn.edu (F.E.P.); larga002@umn.edu (D.A.L.)

**Keywords:** doxorubicin, tumor, inflammation

## Abstract

Doxorubicin (DOX) is one of the most widely used chemo-therapeutic agents in pediatric oncology. DOX elicits an inflammatory response in multiple organs, which contributes to DOX-induced adverse effects. Cancer itself causes inflammation leading to multiple pathologic conditions. The current study investigated the inflammatory response to DOX and tumors using an EL4-lymphoma, immunocompetent, juvenile mouse model. Four-week old male C57BL/6N mice were injected subcutaneously with EL4 lymphoma cells (5 × 10^4^ cells/mouse) in the flank region, while tumor-free mice were injected with vehicle. Three days following tumor implantation, both tumor-free and tumor-bearing mice were injected intraperitoneally with either DOX (4 mg/kg/week) or saline for 3 weeks. One week after the last DOX injection, the mice were euthanized and the hearts, livers, kidneys, and serum were harvested. Gene expression and serum concentration of inflammatory markers were quantified using real-time PCR and ELISA, respectively. DOX treatment significantly suppressed tumor growth in tumor-bearing mice and caused significant cardiac atrophy in tumor-free and tumor-bearing mice. EL4 tumors elicited a strong inflammatory response in the heart, liver, and kidney. Strikingly, DOX treatment ameliorated tumor-induced inflammation paradoxical to the effect of DOX in tumor-free mice, demonstrating a widely divergent effect of DOX treatment in tumor-free versus tumor-bearing mice.

## 1. Introduction

Doxorubicin (DOX), also known as Adriamycin, is one of the most widely used chemotherapeutic agents in pediatric clinical oncology. Despite its effectiveness in a wide range of cancers including lymphoma, leukemia, and other pediatric cancers, DOX clinical utility is limited due to its multiple organ toxicities. In addition to its marked cardiotoxicity [1], DOX has been demonstrated to cause nephrotoxicity [2,3,4,5] and hepatotoxicity [6]. Nearly 50% of pediatric cancer patients receive DOX as part of their treatment protocol [7]. Due to effective cancer treatments, including DOX, the 5 year survival rate of children diagnosed with cancer has increased from less than 60% in the 1970s to more than 80% now [8,9]. This leads to a large population of childhood cancer survivors (CCSs), estimated to be around 400,000. Unfortunately, CCSs are at high risk of developing long-term adverse effects due to cancer treatment and/or cancer itself.

DOX has been shown to trigger systemic inflammation [10,11,12] and an inflammatory response in multiple organs including the heart [13], kidney [14], and liver [15]. This inflammatory response has been shown to promote DOX-induced adverse effects [16,17]. The majority of these studies reporting DOX-induced inflammation have been conducted in tumor-free animal models which do not mimic the clinical scenario in which pediatric cancer patients receive DOX treatment after diagnosis with cancer. Cancer per se has also been shown to cause inflammation contributing to multiple pathologic conditions that may exacerbate DOX-induced toxicities [18,19]. Therefore, there is a critical need to establish clinically relevant animal models to investigate the mechanisms of DOX-induced multiple organ toxicities in young, tumor-bearing mice to study the interplay between DOX treatment and cancer itself to cause such toxicities.

In the current study, we investigated the inflammatory response to DOX and the tumor in a clinically relevant syngeneic EL4-lymphoma, immunocompetent, juvenile mouse model. This model is well established in cancer research [20] and is clinically relevant to pediatric cancer patients since lymphoma is one of the most common types of pediatric cancer [21]. The use of syngeneic mice is critical for studying the inflammatory response to both DOX and the tumor due to the critical role of the immune system in the pathogenesis of inflammation [22]. The mice age was 5 weeks upon DOX administration, which is equivalent to the adolescence age of 10–12 years in humans [23]. Additionally, the administered DOX doses are clinically relevant to the doses received by pediatric cancer patients [24]. We first determined the effects of DOX on cardiac function in tumor-free and tumor-bearing mice, since DOX-induced cardiotoxicity is considered the most clinically relevant adverse effect of DOX, among other multiple organ toxicities. Thereafter, we determined the effect of DOX, the tumor, and their combination on several inflammatory markers in the heart, liver, and kidney in addition to serum markers of systemic inflammation. We found that the presence of EL4 tumor elicited a strong inflammatory response in multiple organs. Strikingly, DOX treatment ameliorated tumor-induced inflammation paradoxical to the previously reported effect of DOX in tumor-free mice.

## 2. Results

### 2.1. Effects of DOX on Body Weight and Tumor Growth

**Figure 1:**
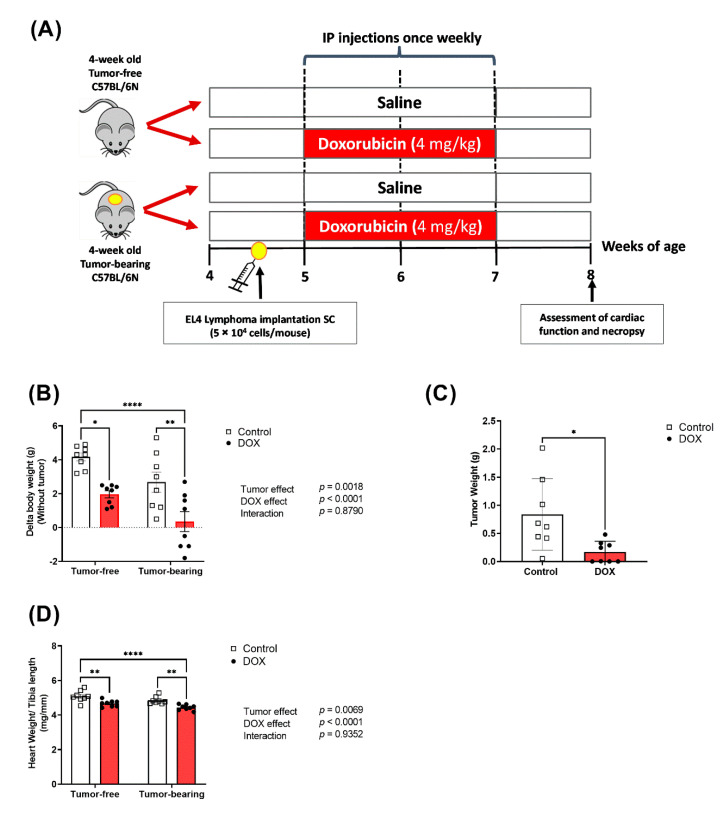
Model schematic diagram and body weight data. (**A**) Scheme of the experimental study design to investigate the effects of low dose DOX in EL4 lymphoma tumor-bearing C57BL/6N juvenile mice. One week following administration of intraperitoneal (IP) DOX (4 mg/kg/week) or equivalent volume of saline for 3 weeks, the final body weight with and without tumors was recorded, and (**B**) the change from baseline body weight was calculated (*n* = 8 per group); (**C**) tumors were harvested and weighed (*n* = 8 per group); (**D**) hearts were harvested from both tumor-free and EL4 lymphoma tumor-bearing mice one week following the administration of 4 mg/kg/week of DOX or equivalent volume of sterile saline for 3 weeks and hearts weights (mg) were measured and normalized to the tibial length (mm) (HW/TL) (*n* = 8 per group). Two-way ANOVA table demonstrates DOX, tumor, and interaction effects. Statistical significance of pairwise comparisons was determined using two-way ANOVA with Tukey’s post hoc analysis. Tumor weights were compared using unpaired Student’s two-tailed *t*-test. (* *p* < 0.05, ** *p* < 0.01, and **** *p* < 0.0001).

The current study was established in juvenile mice to mimic the clinical scenario in pediatric cancer patients as described in Figure 1A. No significant morbidity or mortality was observed in either tumor-free or tumor-bearing mice following exposure to DOX (4 mg/kg/week for 3 weeks), which is in agreement with our previous study using the same dose [25]. However, juvenile exposure to DOX resulted in a significantly reduced body weight gain compared to saline-treated mice (Figure 1B). To assess the anticancer effect of DOX in this EL4 lymphoma model, we measured the tumor weights following mice necropsy. Chronic DOX administration in tumor-bearing mice significantly inhibited the EL4 tumor growth, demonstrating the anticancer effect of this dosage regimen of DOX (Figure 1C).

### 2.2. Effect of DOX Administration on Cardiac Function and Morphometry in Tumor-Free and Tumor-Bearing Mice

**Figure 2:**
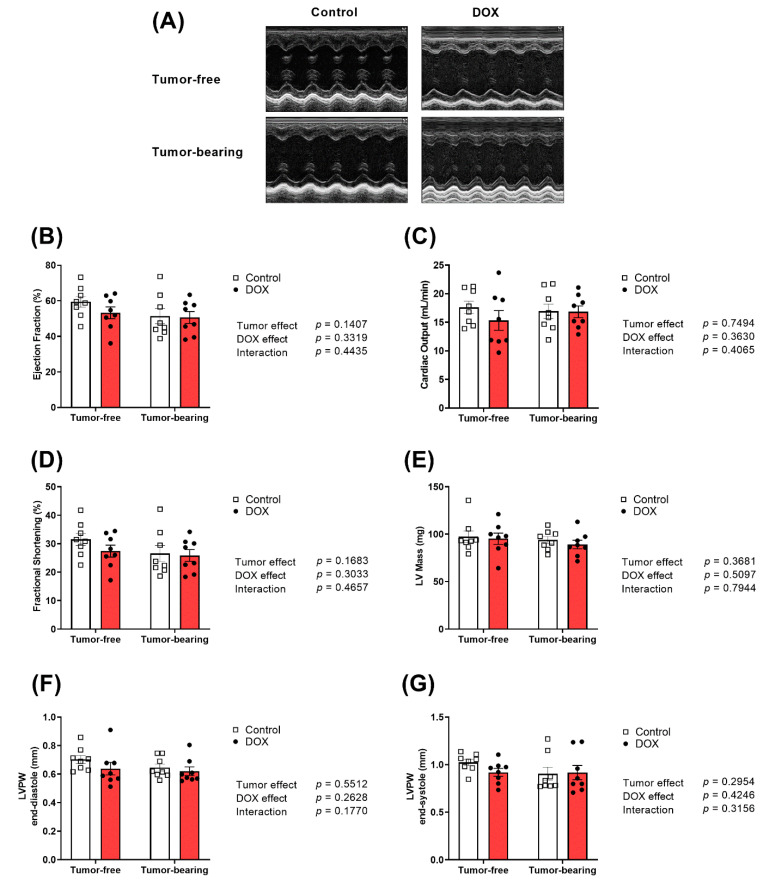
Chronic administration of DOX (4 mg/kg/week) for 3 weeks causes a similar cardiac response in tumor-free and tumor-bearing juvenile mice. Tumor-free and EL4 lymphoma tumor-bearing juvenile C57BL/6N male mice were administered intraperitoneal DOX (4 mg/kg/week) or saline for 3 weeks. Cardiac function was assessed by transthoracic echocardiography one week following the last injection (*n* = 8 per group). (**A**) Representative images from parasternal short axis view of the heart acquired in M-Mode. Effects of DOX and the tumor on (**B**) ejection fraction, (**C**) cardiac output, (**D**) fractional shortening, (**E**) left ventricular (LV) mass, (**F**) LV posterior wall during diastole, and (**G**) LV posterior wall during systole. Values are shown as means ± SEM. Two-way ANOVA was used to determine the main effects of DOX, tumor, and interaction. Statistical significance of pairwise comparisons was determined using two-way ANOVA with Tukey’s post hoc analysis.

DOX caused cardiac atrophy as demonstrated by a significant decrease in the heart weight to tibia length (HW/TL) (Figure 1D). Remarkably, cardiac atrophy caused by DOX was augmented in tumor-bearing mice (Figure 1D). The effects of DOX, tumor, and the combination of both on cardiac function and morphology were assessed using trans-thoracic echocardiography. Representative M-Mode images from each group are displayed in Figure 2A. Neither DOX nor the tumor caused significant changes in ejection fraction (Figure 2B), cardiac output (Figure 2C), fractional shortening (Figure 2D), left ventricular (LV) mass (Figure 2E), LV posterior wall thickness in diastole (LVPW;d) (Figure 2F), or LV posterior wall thickness in systole (LVPW;s) (Figure 2G).

### 2.3. Effect of DOX Administration on Gene Expression of Inflammatory Markers in the Heart of Tumor-Bearing and Tumor-Free Mice

**Figure 3:**
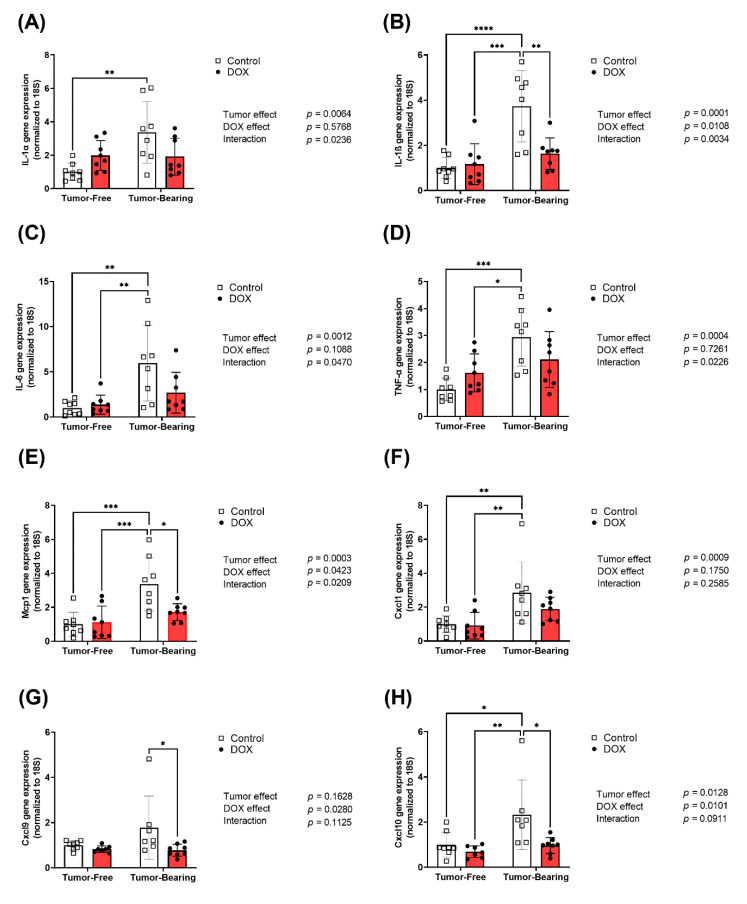
Chronic administration of DOX (4 mg/kg/week) for 3 weeks abrogates tumor-induced upregulation of inflammatory markers in the heart. Hearts were harvested from both tumor-free and EL4 lymphoma tumor-bearing juvenile C57BL/6N mice one week following the administration of 4 mg/kg/week of DOX or equivalent volume of sterile saline for 3 weeks (*n* = 7–8 per group). Following the extraction of total RNA, cardiac mRNA expression of (**A**) IL-1α, (**B**) IL-1β, (**C**) IL-6, (**D**) TNF-α, (**E**) Mcp-1, (**F**) Cxcl1, (**G**) Cxcl9, and (**H**) Cxcl10 were determined by real-time PCR. Values were normalized to 18S rRNA and expressed relative to saline-treated tumor-free mice. Values are shown as means ± SEM. Two-way ANOVA table demonstrates DOX, tumor, and interaction effects on the mRNA expression. Statistical significance of pairwise comparisons was determined using two-way ANOVA with Tukey’s post hoc analysis (* *p* < 0.05, ** *p* < 0.01, *** *p* < 0.001, and **** *p* < 0.0001).

Hearts were harvested from both tumor-free and EL4 lymphoma tumor-bearing juvenile C57BL/6N mice one week following the administration of 4 mg/kg/week of DOX or equivalent volume of sterile saline for 3 weeks (*n* = 8 per group). Following the extraction of total RNA, cardiac mRNA expression of inflammatory markers interleukin-1 alpha (IL-1α), interleukin-1 beta (IL-1β), interleukin-6 (IL-6), tumor necrosis factor-alpha (TNF- α), inflammatory chemokines monocyte chemoattractant protein-1 (Mcp-1), and C-X-C motif chemokines (Cxcl1, Cxcl9, and Cxcl10) were determined by real-time PCR. The gene expression of multiple inflammatory markers in the heart was significantly upregulated in tumor-bearing mice as compared to tumor-free mice, including the pro-inflammatory cytokines IL-1α (Figure 3A), IL-1β (Figure 3B), IL-6 (Figure 3C), and TNF- α (Figure 3D) and the inflammatory chemokines Mcp-1 (Figure 3E), Cxcl1 (Figure 3F), and Cxcl10 (Figure 3H). While DOX administration in tumor-free mice did not result in significant changes in the expression of inflammatory markers in the heart, DOX administration significantly abrogated the tumor-induced upregulation of IL-1β (Figure 3B), Mcp-1 (Figure 3E), Cxcl9 (Figure 3G), and Cxcl10 (Figure 3H). Importantly, two-way ANOVA demonstrated a significant interaction effect in all markers except Cxcl1, Cxcl9, and Cxcl10, demonstrating the divergent effects that DOX has on cardiac inflammatory markers in tumor-bearing compared to tumor-free mice.

### 2.4. Effect of DOX Administration on Gene Expression of Inflammatory Markers in the Liver of Tumor-Free and Tumor-Bearing Mice

**Figure 4:**
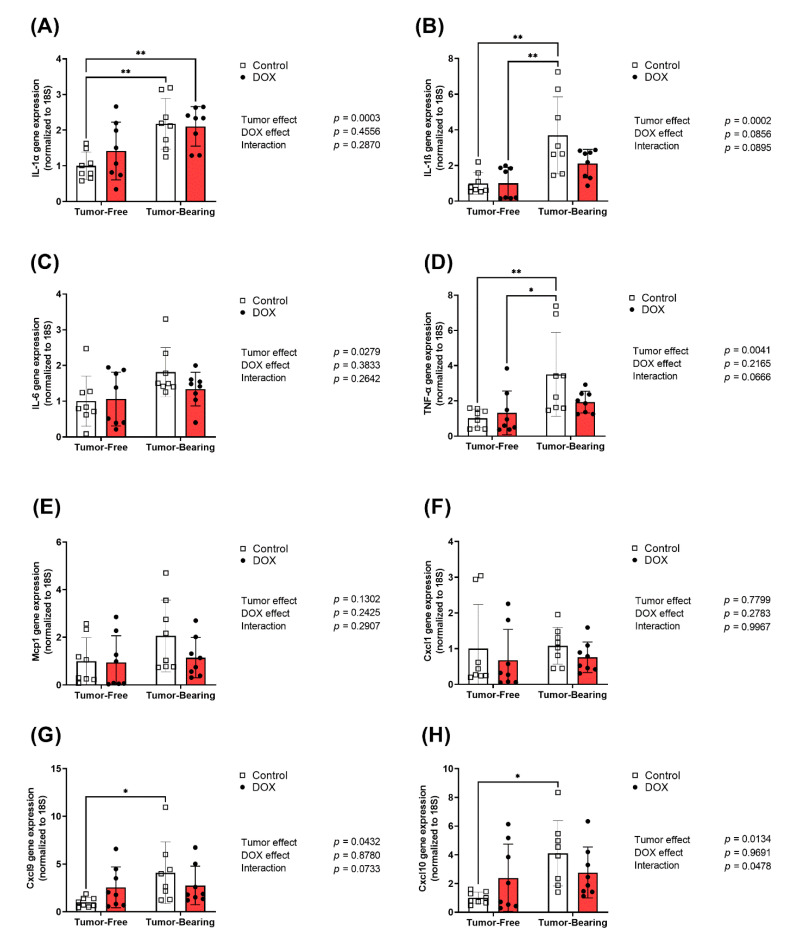
Chronic administration of DOX (4 mg/kg/week) for 3 weeks abrogates tumor-induced upregulation of inflammatory markers in the liver. Livers were harvested from both tumor-free and EL4 lymphoma tumor-bearing juvenile C57BL/6N mice one week following the administration of 4 mg/kg/week of DOX or equivalent volume of sterile saline for 3 weeks (*n* = 8 per group). Following the extraction of total RNA, hepatic mRNA expression of (**A**) IL-1α, (**B**) IL-1β, (**C**) IL-6, (**D**) TNF-α, (**E**) Mcp-1, (**F**) Cxcl1, (**G**) Cxcl9, and (**H**) Cxcl10 were determined by real-time PCR. Values were normalized to 18S rRNA and expressed relative to saline-treated tumor-free mice. Values are shown as means ± SEM. Two-way ANOVA table demonstrates DOX, tumor, and interaction effects on the mRNA expression. Statistical significance of pairwise comparisons was determined using two-way ANOVA with Tukey’s post hoc analysis (* *p* < 0.05, ** *p* < 0.01).

Livers were harvested from both tumor-free and EL4 lymphoma tumor-bearing juvenile C57BL/6N mice one week following the administration of 4 mg/kg/week of DOX or equivalent volume of sterile saline for 3 weeks (*n* = 8 per group). Following the extraction of total RNA, hepatic mRNA expression of inflammatory markers IL-1α, IL-1β, IL-6, TNF-α, Mcp-1, Cxcl1, Cxcl9, and Cxcl10 were determined by real-time PCR. Tumor-bearing mice had significantly elevated hepatic expression of the pro-inflammatory cytokines IL-1α (Figure 4A), IL-1β (Figure 4B), TNF-α (Figure 4D), Cxcl9 (Figure 4G), and Cxcl10 (Figure 4H) compared to tumor-free mice. Although less remarkable, two-way ANOVA demonstrates a significant effect of the tumor on the hepatic gene expression of IL-6 (Figure 4C). Two-way ANOVA demonstrated a significant interaction effect between the tumor and DOX in Cxcl10 (Figure 4H). DOX administration reduced tumor-induced upregulation of IL-1β and TNF-α (Figure 4B,D). Neither DOX nor the tumor had a significant effect on the hepatic expression of inflammatory chemokines Mcp-1 and Cxcl1 (Figure 4E,F).

### 2.5. Effect of DOX Administration on Gene Expression of Inflammatory Markers in the Kidney of Tumor-Free and Tumor-Bearing Mice

**Figure 5:**
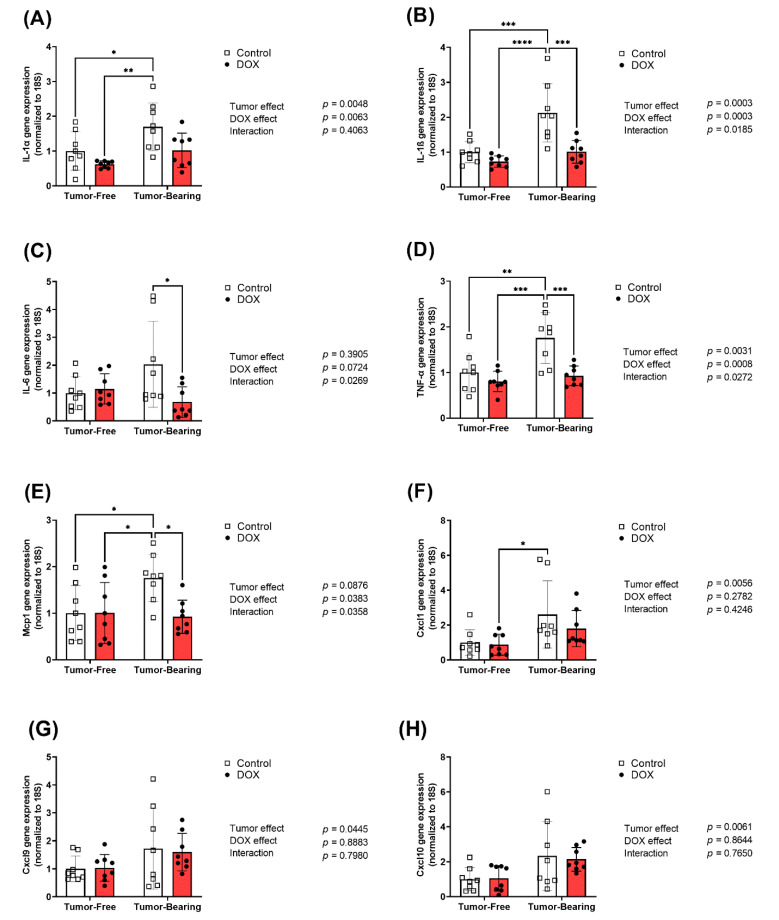
Chronic administration of DOX (4 mg/kg/week) for 3 weeks abrogates tumor-induced upregulation of inflammatory markers in the kidney. Kidneys were harvested from both tumor-free and EL4 lymphoma tumor-bearing juvenile C57BL/6N mice one week following the administration of 4 mg/kg/week of DOX or equivalent volume of sterile saline for 3 weeks (*n* = 8 per group). Following the extraction of total RNA, renal mRNA expression of (**A**) IL-1α, (**B**) IL-1β, (**C**) IL-6, (**D**) TNF-α, (**E**) Mcp-1, (**F**) Cxcl1, (**G**) Cxcl9, and (**H**) Cxcl10 were determined by real-time PCR. Values were normalized to 18S rRNA and expressed relative to saline-treated tumor-free mice. Values are shown as means ± SEM. Two-way ANOVA table demonstrates DOX, tumor, and interaction effects on the mRNA expression. Statistical significance of pairwise comparisons was determined using two-way ANOVA with Tukey’s post hoc analysis (* *p* < 0.05, ** *p* < 0.01, and *** *p* < 0.001, **** *p* < 0.0001).

Kidneys were harvested from both tumor-free and EL4 lymphoma tumor-bearing juvenile C57BL/6N mice one week following the administration of 4 mg/kg/week of DOX or equivalent volume of sterile saline for 3 weeks (*n* = 8 per group). Following the extraction of total RNA, renal mRNA expression of inflammatory markers IL-1α, IL-1β, IL-6, TNF-α, Mcp-1, Cxcl1, Cxcl9, and Cxcl10 were determined by real-time PCR. In line with the observed changes in the heart and the liver, tumor-bearing mice had significantly increased renal expression of the pro-inflammatory cytokines IL-1α (Figure 5A), IL-1β (Figure 5B), TNF-α (Figure 5D), and Mcp-1 (Figure 5E) compared to tumor-free mice. Additionally, two-way ANOVA demonstrates a significant effect of the tumor on the renal gene expression of Cxcl1 (Figure 5F), Cxcl9 (Figure 5G), and Cxcl10 (Figure 5H). DOX administration in tumor-bearing mice significantly ameliorated the tumor-induced upregulation of IL-1β (Figure 5B), IL-6 (Figure 5C), TNF-α (Figure 5D), and Mcp-1 (Figure 5E). Within those markers, two-way ANOVA also demonstrated a significant interaction between DOX and the tumor demonstrating the different effects that DOX has on renal inflammatory markers in tumor-bearing compared to tumor-free mice.

### 2.6. Effect of DOX Administration on Histopathological Features in the Heart, Liver, and Kidney of Tumor-Free and Tumor-Bearing Mice

**Figure 6:**
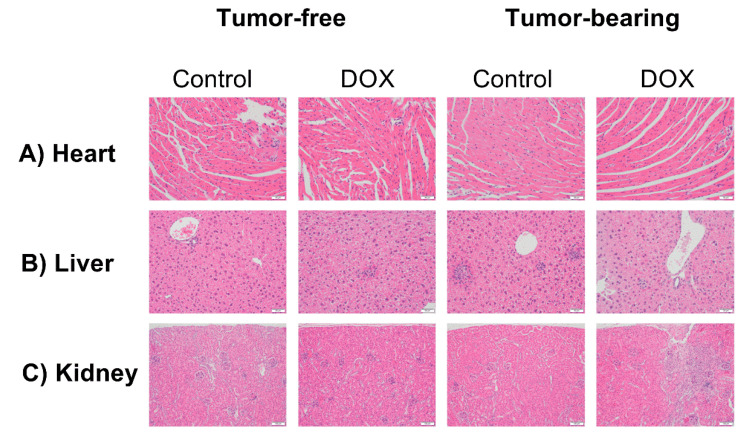
Histopathological evaluation of tumor-free and EL4 lymphoma tumor-bearing juvenile C57BL/6N mice euthanized one week following the administration of 4 mg/kg/week of DOX or equivalent volume of sterile saline for 3 weeks. Representative images from hematoxylin and eosin (HE)-stained sections of (**A**) heart, (**B**) liver, and (**C**) kidney are shown. Scale bar = 50 µM (A and B) and 100 µM (C).

Hearts, livers, and kidneys were harvested from one cohort of tumor-free and EL4 lymphoma tumor-bearing juvenile C57BL/6N mice one week following the administration of 4 mg/kg/week of DOX or equivalent volume of sterile saline for 3 weeks (*n* = 4 per group). Histopathological evaluation demonstrated no major pathological changes in the heart (Figure 6A). Only minimal inflammatory cell infiltration, demonstrated as rare foci of minimally cellular mixed (mononuclear and neutrophilic) inflammation, was observed in the liver tissues harvested from DOX-treated tumor-free (three out of four mice), saline-treated tumor-bearing (two out of four mice), and DOX-treated tumor-bearing mice (three out of four mice) (Figure 6B). Histopathological evaluation of the kidneys demonstrated no major pathological changes in all groups except DOX-treated tumor-bearing mice (Figure 6C), with two out of the four mice in this group demonstrating regions of full thickness tubular regeneration, fibrosis, and mild interstitial inflammation affecting approximately 5% of total tissue area.

### 2.7. Divergent Effects of DOX on Serum Inflammatory Cytokines in Tumor-Free Versus Tumor-Bearing Mice

**Figure 7:**
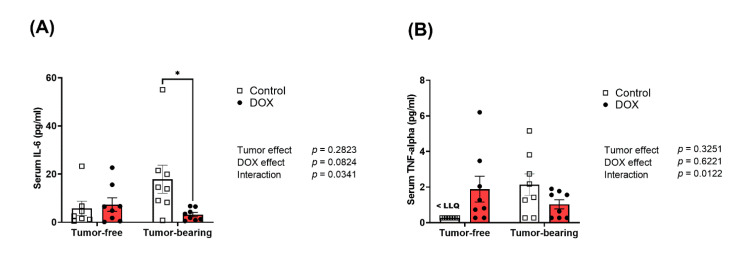
Divergent effects of DOX treatment on the serum level of inflammatory markers in tumor-free versus tumor-bearing mice. Serum was collected from tumor-free and EL4 lymphoma tumor-bearing juvenile C57BL/6N mice one week following the administration of 4 mg/kg/week of DOX or equivalent volume of sterile saline for 3 weeks (*n* = 7–8 per group). Inflammatory markers (**A**) IL-6 and (**B**) TNF-alpha were measured using the Luminex platform. Data are presented as the mean ± SEM. Two-way ANOVA table demonstrates DOX, tumor, and interaction effects. Statistical significance of pairwise comparisons was determined using two-way ANOVA with Tukey’s post hoc analysis (* *p* < 0.05).

We have previously reported that acute DOX administration elicited a systemic inflammatory response in adult C57BL/6N mice [11]. Herein, we determined the effect of chronic DOX administration on serum levels of the inflammatory markers IL-6 and TNF-α, in juvenile tumor-free and tumor-bearing mice. Saline-treated tumor-bearing mice demonstrated a marked, though statistically insignificant, increase in serum IL-6 (Figure 7A) and TNF-α (Figure 7B) compared to tumor-free mice. Importantly, two-way ANOVA demonstrated a significant interaction between DOX administration and the tumor on the serum levels of IL-6 and TNF-α. Indeed, DOX administration normalized tumor-induced upregulation of IL-6 to a similar level as control tumor-free mice (Figure 7A).

## 3. Discussion

Around 10,000 children will be diagnosed with cancer in the United States in 2021 [9]. Thanks to advanced diagnosis, novel treatments, and improved care models, the survival rate has increased dramatically over the past 40 years and has reached around 85% [9]. However, this increased survival comes with a cost. Indeed, childhood cancer survivors have a considerably increased risk for multiple chronic health conditions including premature cardiovascular diseases [26], mainly due to cancer treatment and/or cancer itself. Nearly 50% of pediatric cancer patients receive DOX, a cardiotoxic anthracycline. Although a few tumor-bearing preclinical models studied DOX-induced toxicity in adult animals [27,28,29], most animal models for juvenile DOX cardiotoxicity have been established in tumor-free mice, which precludes the significant role that tumors may play [25,30,31,32]. Importantly, there is only one recent study that described the cardiotoxic effects of DOX in juvenile tumor-bearing mice [33]. Although important, this study used immunocompromised nude mice implanted with human cancer cells. This approach precludes studying the role of the adaptive immune system that is known to be critical in DOX-induced toxicity and inflammation [34]. To study the interaction between DOX- and tumor-induced inflammation, we used EL4 lymphoma syngeneic immunocompetent tumor-bearing mice.

The cardiotoxic effects of DOX are shown to be dependent on the cumulative dose [1]. Therefore, the current treatment protocols usually do not exceed this threshold. Consequently, the rates of severe cardiovascular complications have decreased recently [35]. However, juvenile DOX-induced cardiotoxicity is often characterized in preclinical models using high cumulative doses of DOX that are enough to cause immediate or delayed cardiac dysfunction [36,37,38,39]. We have previously shown that low doses of DOX administered to young mice did not cause immediate cardiac dysfunction but rather a latent (subclinical) cardiotoxicity which can be unmasked by exposure to adult-onset cardiovascular stressors such as high blood pressure [25] or psychosocial stress [40]. Remarkably, these latent cardiotoxic effects are rarely characterized in juvenile animal models. Therefore, in the current study, both tumor-free and tumor-bearing juvenile mice were administered a low-dose DOX regimen (4 mg/kg/week for 3 weeks) as a model for latent DOX cardiotoxicity [25]. This dosage regimen is equivalent to a human dose of 40 mg/m^2^ [41], which is relevant to the low range of clinical doses used to treat hematologic malignancies in pediatric cancer patients [24]. We demonstrate that chronic administration of low doses of DOX inhibited EL4 tumor growth, confirming that this low-dose DOX regimen still offers anticancer effects. This was consistent with a previous study which demonstrated that DOX (6 mg/kg for three doses) resulted in complete tumor regression in the majority of EL4 tumor-bearing mice [42]. It was critical to demonstrate the chemotherapeutic benefit of the low-dose DOX regimen in this mouse model so that it can be used in the future for evaluating potential cardioprotective strategies to confirm that they do not compromise the chemotherapeutic benefit of DOX before translating to the clinical settings.

Consistent with our previous findings, chronic administration of DOX (4 mg/kg/week) for 3 weeks caused a significant decrease in body weight compared to saline-treated mice. Cancer-induced cachexia, one of the complications associated with cancer that occur due to tumor-derived factors, is associated with a decreased body mass. Nevertheless, in the current study, there was no marked difference in body weight gain in saline-treated tumor-bearing mice relative to saline-treated tumor-free mice. This can be explained by EL4 lymphoma cells having been shown to not induce cachexia and being considered non-cachectic cancer cells [43]. DOX caused significant cardiac atrophy without reducing the contractile function of the heart in both tumor-free and tumor-bearing mice. Additionally, DOX-induced cardiac atrophy was augmented in tumor-bearing mice. This is consistent with previous studies showing cardiac atrophy in tumor-bearing mice compared to tumor-free mice [44,45,46] and supported by the clinical evidence of cardiac atrophy in anthracycline-treated childhood cancer survivors [47,48,49,50]. Experimental and clinical evidence suggests that cardiac atrophy caused by low/divided doses of DOX is mediated by DOX-induced cardiomyocyte atrophy [51,52,53], while high doses of DOX may cause cardiac atrophy due to apoptotic and necrotic cell death and loss of cardiomyocytes [54]. Since we used low doses of DOX in the current study, the observed cardiac atrophy in DOX-treated mice is most likely attributed to cardiomyocyte atrophy.

In addition to its cardiotoxicity, DOX itself was shown to trigger systemic inflammation and upregulate multiple inflammatory markers in the heart, kidney, and liver, which can contribute to DOX-induced cardiotoxicity, nephrotoxicity, and hepatotoxicity, respectively [13,14,15,16,17]. In addition to DOX, cancer itself has been shown to trigger an inflammatory response that initially starts within the tumor microenvironment, and then the secreted cytokines can result in a systemic inflammatory response [55], which could also affect multiple distal non-metastatic organs [56]. Clinical studies have shown that childhood cancer survivors have elevated levels of inflammatory cytokines. For example, Ariffin et al. demonstrated that childhood cancer survivors of acute lymphoblastic leukemia have elevated levels of plasma proinflammatory markers [57]. However, there is a paucity of clinical studies that assess the effects of cancer treatment on inflammation in distal organs since human biopsies for these organs are challenging and rare. Considering all of that, we sought to determine the effect of chronic low doses of DOX on the expression of inflammatory markers in tumor-bearing mice in multiple organs including heart, liver, and kidney and systemically in the serum.

Most previously conducted animal studies emphasize how cancer therapeutics promote inflammation or cardiac dysfunction, while the effects of cancer development are not well researched. Intriguingly, cancer itself can affect cardiovascular health through independent signaling pathways other than those triggered by cancer treatment [58]. In a recent clinical study, chemotherapy-naive patients with newly diagnosed lymphoma demonstrated reduced cardiac chamber volumes compared to healthy volunteers [58]. One of the suggested pathways in which cancer could affect the heart is through cancer-induced inflammation [59]. In agreement with that, we demonstrated an increase in the expression of multiple inflammatory markers in the heart in EL4 tumor-bearing mice compared to the tumor-free group. However, the triggered inflammation did not cause pathological alterations in the heart tissue within the timeline of our current experiment. The long-term effect of the observed inflammatory response on cardiac pathology is yet to be determined. Interestingly, DOX administration in tumor-bearing mice attenuated the tumor-induced inflammatory response.

Next, we sought to determine if the interaction between the tumor and DOX on hepatic inflammation would be the same. DOX administration has been shown to trigger inflammation in the liver which can contribute to DOX-induced hepatotoxicity [5,15]. Notably, these studies used acute DOX administration and tumor-free mice. By contrast, our chronic low dose DOX (4 mg/kg/week for 3 weeks) did not induce significant upregulation of inflammatory markers in tumor-free mice. Previous EL4 tumor-bearing mice models demonstrated elevated levels of ALT and AST, which suggests moderate liver damage, due to accumulation of immune cells in the livers [60]. Consistent with these results, we demonstrated an upregulation of hepatic inflammatory markers in tumor-bearing mice compared to the tumor-free group. In agreement with our findings in the heart, DOX administration in tumor-bearing mice attenuated tumor-induced inflammation in the liver. Rare foci of inflammatory cell infiltration were observed in all groups of mice, except for the saline-treated tumor-free mice.

DOX also was shown to increase the expression of proinflammatory markers in the kidneys in previous animal studies which can contribute to DOX-induced nephrotoxicity [14]. However, the chronic low dose of DOX did not significantly alter the expression of inflammatory markers in tumor-free mice. Similar to the heart and liver, we demonstrated an increase in the expression of inflammatory markers in the kidneys of tumor-bearing mice. This was consistent with a previous study that demonstrated that expression of renal inflammatory markers and adhesion molecules are upregulated in kidneys of tumor-bearing mice in a phenotype that is similar to acute renal failure [56]. Importantly, tumor-induced upregulation of inflammatory markers was abrogated by DOX administration. Intriguingly, pathological changes were detected only in kidney tissues of DOX-treated tumor-bearing mice, an observation that warrants further investigation in future studies.

Finally, we wanted to investigate the effects of DOX and the tumor on systemic inflammation. DOX itself has been shown in previous models to induce systemic inflammation [11,61]. However, the DOX regimen used in our study did not cause a significant increase in serum inflammatory markers in tumor-free mice. In agreement with a previous EL4 tumor-bearing mouse model [60], we demonstrate that EL4 lymphoma induced a systemic upregulation of serum proinflammatory cytokines. Paradoxically, DOX administration in tumor-bearing mice ameliorated the tumor-induced systemic inflammation, an effect that may be attributed to suppression of tumor growth by DOX. Although this observation is in contrast to other studies demonstrating DOX-induced inflammation in tumor-free mice, it concurs with studies conducted in tumor-bearing mice [59,62], suggesting widely divergent effects of DOX treatment in tumor-free versus tumor-bearing mice.

## 4. Materials and Methods

### 4.1. Animals

All animal procedures in this study were approved by the Institutional Animal Care and Use Committee (IACUC) at the University of Minnesota (Protocol ID: 1807-36187A). Juvenile (four weeks old) male (*n* = 32) C57BL/6N mice were purchased from Charles River Laboratories (Wilmington, MA, USA). All mice were given an acclimation period of three days and housed in groups of 4 mice per cage and maintained under standard specific pathogen-free (SPF) conditions. Mice were given food and water ad libitum in a 14 h light/10 h dark cycle at 21 ± 2 °C.

A graphical representation of the study design is shown in Figure 1A. At 4.5 weeks of age, to establish the tumor-bearing mouse model, mice were given a single subcutaneous injection in the flank region containing 5 × 10^4^ EL4 lymphoma cells suspended in sterile PBS (*n* = 16, tumor-bearing group) or an equivalent volume of sterile PBS (*n* = 16, tumor-free group). Three days after tumor inoculation, tumor-free and tumor-bearing mice were randomly assigned to receive an intraperitoneal injection of DOX (4 mg/kg/week for 3 weeks) or an equivalent volume of sterile saline (control group), such that we had four groups including saline-treated tumor-free, DOX-treated tumor-free, saline-treated tumor-bearing, and DOX-treated tumor-bearing (*n* = 8 per group). To ensure the reproducibility of our findings, the experiment was performed in two cohorts of mice (*n* = 4 per group in each cohort). Animal weights were assessed weekly. One week after the last dose of DOX or saline, mice from all groups were humanely euthanized by decapitation under isoflurane anesthesia. Terminal blood was collected, and multiple organs were harvested including heart, liver, kidney, and tumor (from the tumor-bearing group). Organs were rinsed in ice-cold phosphate-buffered saline (PBS), flash-frozen in liquid nitrogen, and stored at −80 °C until further analysis.

### 4.2. EL4 Cell Culture and Reagents

Murine EL4 lymphoma cells were kindly provided by Dr. David Largaespada (University of Minnesota, Minneapolis, MN, USA). Suspended EL4 cells were cultured at 37 °C in RPMI-1640 medium (Corning 10-040-CV) supplemented with 10% (*v*/*v*) fetal bovine serum, 100 U/mL penicillin, and 100 μg/mL streptomycin in a humidified atmosphere containing 5% CO_2_. EL-4 cells were harvested for injection following 2–3 passages. Cells were pelleted at 300 × g and washed 2 times with sterile PBS. Cells were reconstituted to a concentration of 500,000 cells/mL as determined by cell count, and cellular health was qualitatively determined visually.

### 4.3. Echocardiography

The effect of the tumor and DOX administration on cardiac function was evaluated using transthoracic echocardiography performed one week following the last DOX injection (*n* = 8 per group). Echocardiography was conducted using the Vevo 2100 system (VisualSonics, Inc., Toronto, ON, Canada) equipped with an MS400 transducer as previously described [63]. Anesthesia was induced using 3% isoflurane and was maintained with 1–2% isoflurane during the procedure. The anesthetic level was assessed by toe pinch and monitoring respiratory rate. Mice were held in a supine position on a heated physiologic monitoring stage during the procedure. M-Mode was used to acquire parasternal short-axis images of the left ventricle at the level of the papillary muscles. Throughout 3–4 cardiac cycles, the endocardial and epicardial boundaries were manually traced, and cardiac function and morphometric parameters were measured using the VisualSonics cardiac measurement package of the Vevo 2100.

### 4.4. RNA Extraction and Real-Time PCR

Total RNA was extracted from 20 mg frozen heart, liver, kidney, and tumor tissues using 300 μL Trizol reagent (Life Technologies, Carlsbad, CA, USA) according to the manufacturer’s instructions. Then, RNA concentrations were quantified using a Nanodrop 8000 spectrophotometer (Thermo Fisher Scientific, Wilmington, DE, USA). Thereafter, first-strand cDNA was synthesized from 1.5 μg total RNA using the high-capacity cDNA reverse-transcription kit (Applied Biosystems, Foster City, CA, USA) according to manufacturer’s instructions. To measure specific mRNA expression, real-time polymerase chain reaction (PCR) was carried out using SYBR Green (Applied Biosystems, Foster City, CA, USA) in 384-well optical reaction plates and performed on an QuantStudio 7 instrument (Applied Biosystems, Foster City, CA, USA). PCR reactions were performed in a final volume of 20 μL reaction mix consisting of 1 μL cDNA sample, 0.025 μL 30 μM forward primer, 0.025 μL 30 μM reverse primer, 10 μL SYBR Green Universal Mastermix (Life Technologies, Carlsbad, CA, USA), and 8.95 μL of nuclease-free water. Thermocycling amplification conditions were performed as follows: 95 °C for 10 min, then 40 PCR cycles of denaturation at 95 °C for 15 s, and annealing/extension at 60 °C for 1 min. The primers selected in the current study were checked with the Primer-BLAST online tool and are listed in Table 1. Melting curve analysis was implemented to ensure the specificity of the primers used and the purity of the final PCR product. The ΔΔCt method was used to identify relative mRNA expression, after normalizing to 18S ribosomal RNA (18S rRNA). Gene expression is reported relative to the control tumor-free group. PCR experiments were performed by lab personnel who were blinded for the experimental groups.

### 4.5. Histopathology

Following mice necropsy, LV heart, liver, and kidney sections from all groups were collected (*n* = 4 per group), fixed in 10% neutral buffered formalin and embedded in paraffin. Thereafter, four-micron tissue sections of these organs were stained with hematoxylin and eosin (HE) and evaluated for (a) inflammation (distribution, severity, and cell type) and (b) fibrosis by a board-certified veterinary pathologist who was blinded to the experimental groups.

### 4.6. Measurement of Serum Inflammatory Markers

Terminal blood was collected from animals euthanized one week following DOX or saline administration and incubated for 20 min at room temperature to allow blood to clot. Thereafter, samples were centrifuged at 2000× g for 30 min at 4 °C; serum was collected and stored at −80 °C until use. Serum samples were analyzed by the Cytokine Reference Laboratory (University of Minnesota, Minneapolis, MN, USA) for mouse-specific IL-6 and TNF-α using the Luminex platform and conducted as a multiplex as previously described [11]. The magnetic bead set (catalog LXSAMSM-03) was purchased from R&D Systems (Minneapolis, MN, USA). The samples were analyzed according to the manufacturer’s guidelines. Each sample was treated with fluorescent color-coded beads coated with a particular capture antibody. Biotinylated detection antibody was added after incubation and washing, followed by phycoerythrin-conjugated streptavidin. A Luminex instrument (Bioplex 200, Bio-Rad Laboratories, Inc., Hercules, CA, USA) was used to read the beads. Values were interpolated from 5-parameter equipped standard curves after running samples in duplicate. Measurement of serum cytokines was performed by lab personnel who were blinded for the experimental groups.

### 4.7. Statistical Analysis

All data analysis was performed using GraphPad Prism software (Version 9.0, La Jolla, CA, USA). Data are presented as individual data points and their mean ± standard errors of the mean (SEM). Ordinary two-way analysis of variance (ANOVA) followed by Tukey’s multiple comparison post hoc analysis was used to compare between different tumor models and treatment groups. A *p* value of < 0.05 was selected to demonstrate statistical significance.

## 5. Conclusions

In conclusion, our present study demonstrates that the presence of EL4 tumor elicits a strong inflammatory response that can be attenuated by DOX-induced suppression of tumor growth. Additionally, characterizing DOX toxicity in this clinically relevant model emphasizes the divergent effects that DOX has in tumor-free vs. tumor-bearing mice. This highlights the need to develop and use appropriate models to characterize DOX effects so that the findings can be closer to the real clinical scenarios. These preclinical models can be optimum models for testing novel cardioprotective strategies that can be used with chemotherapy, e.g., DOX before translation to pediatric cancer patients with the long-term goal to prevent cancer treatment-induced cardiovascular complications in childhood cancer survivors.

## Figures and Tables

**Table 1:** Primer sequences used in this study.

Gene	Forward Primer (5′–3′)	Reverse Primer (3′–5′)	Ref.
*Cxcl1*	CTGGGATTCACCTCAAGAACATC	CAGGGTCAAGGCAAGCCTC	[64]
*Cxcl9*	ATCTTCCTGGAGCAGTGTGGAGTT	AGGGATTTGTAGTGGATCGTGCCT	[65]
*Cxcl10*	ATATCGATGACGGGCCAGTGAGAA	AATGATCTCAACACGTGGGCAGGA	[65]
*IL-1α*	CGCTTGAGTCGGCAAAGAAAT	TGGCAGAACTGTAGTCTTCGT	[64]
*IL-1β*	TCCTCGGCCAAGACAGGTCGCT	CCCCCACACGTTGACAGCTAGGT	[66]
*IL-6*	CCAGAGATACAAAGAAATGATGG	ACTCCAGAAGACCAGAGGAAAT	[67]
*MCP-1*	GCATCCACGTGTTGGCTCA	CTCCAGCCTACTCATTGGGATCA	[68]
*TNF alpha*	CCAGACCCTCACACTCAGATCA	CACTTGGTGGTTTGCTACGAC	[67]
*r18S*	GTAACCCGTTGAACCCCATT	CCATCCAATCGGTAGTAGCG	[69]

## Data Availability

The data presented in this study are contained within the article.
